# Book Reviews

**Published:** 1903-05

**Authors:** 


### BOOK REVIEWS.

Obstetrics. A Textbook for the use of Students and Practitioners. By J. Whitridge Williams, Professor of Obstetrics, Johns Hopkins University; Obstetrician-in-Chief to the Johns Hopkins Hospital; Gynecologist to the Union Protestant Infirmary, Baltimore. Octavo, pp. 867. With 8 colored plates and 630 illustrations in the text. New York and London: D. Appleton & Co. 1903. [Price, $6.00.]

This treatise considers obstetrics in eight sections, in the following order—anatomy, physiology and development of the ovum, physiology of pregnancy, physiology of labor, obstetric surgery, pathology of pregnancy, pathology of labor, and pathology of the puerperium.

The studies of the anatomy of the uterus and its adnexa are unique, and afford the student opportunity for attractive occupation in the acquirement of completer knowledge. The development of the ovum and placenta have never been set forth as well in a work on obstetrics, according to our view, as by Williams. The illustrations showing the structure of the placenta are admirable.

In the section on obstetric surgery, beginning with induction of abortion and accouchement forcé, including forceps and version, Cesarean section and symphysiotomy, destructive operations, and ending with operative procedures which do not aim at delivery, is found quite the most ample handling of operative obstetrics that has yet been published in such a treatise. Here, again, illustrations supplement the text in an instructive fashion.

Williams's dealing with contracted and otherwise deformed pelves, and the management of labor in such conditions, is most satisfactory. Injuries of the birth canal, infection, hemorrhage, and the puerperium are all prepared by one who understands the greatest need of the student, and he tells him in the fewest and plainest words possible what he must know to obtain success in the practice of obstetrics. It is a book made by a clinician, which gives the most advanced exposition of the art and is a distinct addition to obstetric literature.

Much original work has been done in the way of illustration, as well as in the preparation of the material of many chapters, and the whole subject matter is presented in an original manner. The book is a credit to both author and publisher.

---

HUNTINGTON'S ABDOMINAL ANATOMY. The Anatomy of the Human Peritoneum and Abdominal Cavity considered from the Standpoint of Development and Comparative Anatomy. By GEORGE S. HUNTINGTON, M. D., Professor of Anatomy in the College of Physicians and Surgeons, Columbia University, New York City. In one quarto volume of 292 pages, including 300 full-page plates in colors and monochrome, containing 582 figures. Philadelphia and New York: Lea Brothers & Co. 1903. [De luxe edition, gilt top, uncut edges, $10.00 net.]

The study of anatomy, always fascinating, has been conducted on the lines of ultraestheticism by this author, for which he deserves the thanks and congratulations of a large and ever increasing class of students in anatomy. The publication of this work marks a novel departure, both in material and construction, from the well-beaten paths that have been left in the wake of previous teachers, authors, and publishers of anatomical treatises. The book-making art is developed to the highest standard practical for medical works in this handsome *edition de luxe*, in which paper, binding, letter-press, and artistic illustrations combine and blend with exquisite taste and skill. In a book of not quite 300 pages, there are 300 full-page plates, containing 582 figures,—a most remarkable wealth of illustration.

The author disclaims to offer a complete or detailed account of the development of the abdominal cavity and its contents, but aims rather to utilise certain embryological facts in attempting to explain complicated adult conditions encountered. All through the work appears proof of the value of appealing to embryology and comparative anatomy, for aid in elucidating the sometimes difficult and often complicated morphological problems which confront the student, and lie along his pathway in studying the anatomy of the human adult.

In view of the improved surgery of the liver and gall-bladder, Huntington's studies of the development of the liver will prove of interest, particularly as surgeons are constantly offering proof that the gall-bladder is not an essential to healthy life. Therefore, when this author asserts that the biliary bladder (p. 144) is only a modified portion of the hepatic duct, he offers proof in support of its nonessential character. For similar reasons the parts relating to the intestinal tract, the cecum, and the vermiform appendix will attract the attention of surgeons. It will stimulate further studies in the direction taken and in the field mapped out by this author. Huntington's description of the changes in the position of the cecum and appendix and especially the schematic drawings, illustrating the stages in the rotation of the intestinal canal and descent of the cecum, are full of interest.

The entire work reflects the greatest honor upon Professor Huntington and on Columbia University. It is difficult to understand, however, why in such a masterful treatise the ancient spelling of such words as cecum and fetal should be adhered to. The elimination of the diphthong æ in these and similar words, is in keeping with the teaching of the best modern philologists.

Surgical Anatomy and Operative Surgery. For Students and Practitioners. By John J. McGrath, M. D., Professor of Surgical Anatomy and Operative Surgery at the New York Post-Graduate Medical School; Visiting Surgeon to the Harlem Hospital, and Assisting Visiting Surgeon to the Columbus Hospital, New York. Illustrated with 227 illustrations, including colors and half-tones. Pages xiv–359. Royal octavo. Philadelphia: F. A. Davis Company. 1903. [Extra cloth, $4.00 net; sheep or half-Russia, $5.00 net, delivered.]

In this book is found a blending of anatomy and surgery in a convenient form for both undergraduate and surgeon. Surgical anatomy and operative surgery go hand in hand throughout the work, a plan that is based upon the well-established fact that a knowledge of the one is necessary to the proper study and successful practice of the other.

It is illustrated largely by diagrammatic drawings, which the author has found the most satisfactory method for teaching purposes, and the whole arrangement of the treatise follows the plan adopted by him in his courses at the Post-Graduate Medical School.

The volume is constructed on new lines, and will be criticised by the ultra conservatives perhaps; nevertheless, it is a useful and instructive book, in spite of its eccentricities. It has made an attempt to introduce the new spelling, by dropping the final e in *cocaine*, for example; but if it insists upon this, why should it not also expel the final e from *muriate?* Under the head "Congenital deformities of the face" the author presents a lesson in embryology, which is somewhat unique, but instructive. The work is commendable and shows thorough knowledge of the subjects dealt with by the author.

Culbreth's Materia Medica and Pharmacology. Third edition. A Manual of Materia Medica and Pharmacology. Comprising all Organic and Inorganic Drugs, which are and have been official in the United States Pharmacopeia, together with important Allied Species and Useful Synthetics. By David M. R. Culbreth, M. D., Professor of Botany, Materia Medica, and Pharmacognosy in the Maryland College of Pharmacy, Professor of Materia Medica and Pharmacognosy in the University of Maryland Medical and Dental Schools, Baltimore. Third edition, enlarged and thoroughly revised. In one octavo volume of 905 pages, with 473 illustrations. Philadelphia and New York: Lea Brothers & Co. 1903. [Cloth, $4.75 net.]

This work has rapidly grown into favor as a textbook, it being something more than a manual, as modestly claimed in its title. This revision has taken up the proposed official list of drugs and pharmaceutical preparations, determined upon by the committee charged with revising the pharmacopeia soon to issue. In this way the author has been enabled not only to improve the subject-matter of former editions, but to give new material of interest and importance; also to compile the recapitulation tables in accordance with the official list.

The author has devoted considerable space to the pronunciation of troublesome words, and has paid attention to accentuation, making the work conform to modern philology,—very important points for students, who often find them perplexing.

The literature of materia medica and its cognate topics has been enriched to a considerable degree in late years, but there has been no more conscientious or instructive contribution than Culbreth's book; hence it affords a real pleasure to the reviewer to commend it both to undergraduate students and physicians.

---

Clinical Treatises on the Pathology and Therapy of Disorders of Metabolism and Nutrition. By Prof. Dr. Carl von Noorden, Physician-in-chief to the City Hospital, Frankfort a. M. Authorized American edition translated under the direction of Boardman Reed, M. D., Professor of Diseases of the Gastrointestinal Tract, Hygiene and Climatology, Department of Medicine, Temple College; Physician to the Samaritan Hospital, Philadelphia. Part II, Nephritis. Duodecimo, pp. 112. New York: E. B. Treat & Company. 1903. [Price, $1.00.]

The diseases of the kidney, both acute and chronic, are becoming more and more understood with each succeeding year, and as knowledge increases in this direction there is unfolded a new chart of a field beyond, sometimes more important to explore than its predecessor. The lesson is constantly being taught, with increasing insistence, that diet is the all-important factor in controlling or curing nephritic disease.

This essay will do much to strengthen the argument for more careful dietetic care and for stringent therapeutic measures. It is original in many respects of its treatment of the subject, and will repay well most attentive reading. It is one of the best dressed monographs we have seen in many a day.

---

Transactions of the American Surgical Association. Twentieth annual meeting, held at Albany, June 3–5, 1902. Richard H. Harte, M. D., Recorder of the Association. Philadelphia: William J. Dornan, Printer. 1902.

This volume constitutes a full record of the proceedings of this association at its twentieth annual meeting, but nowhere in the book does it appear where the meeting was held. It would not seem objectionable to record this fact in each volume, and even it might become of importance to have it known.

The group of papers herein published comprise a valuable contribution to the literature of surgery and should be in the hands of every teacher of the topic. They give, in most instances, the personal experience of prominent American surgeons upon technical subjects or points of practice, and will serve as a guide in similar conditions whenever they arise.

We cannot refer to each paper in detail but desire to invite particular attention to one by Rudolph Matas, of New Orleans, relating to the radical cure of aneurism based upon arteriorrhaphy, in which he gives the technic with illustrations, and reports four cases successfully operated by the method. Dr. Matas gives a full account of the operation and refers to Dr. J. B. Murphy's paper on dealing with aneurisms by suture, published in 1897. Dr. Matas, himself, makes a notable contribution to the subject in this communication which will attract the attention of surgeons.

---

Transactions of the Twenty-fourth Annual Meeting of the American Laryngological Association, held at Boston, Mass., May 26, 27 and 28, 1902. James E. Newcomb, M. D., Secretary. New York: Rooney & Otten Printing Co. 1902.

The meeting of which this book contains the proceedings was held at Boston under the presidency of John W. Harlow. It contains an unusual number of scientific papers, principally of interest to the laryngologist. There, are, however, a few papers of general interest, among them one on foreign bodies in the bronchus, one on incised wounds of the larynx, two on hay fever, one on an epidemic of non-suppurative cervical adenitis, and one on precautions to be observed in the use of suprarenal extract in the nose. The latter is a short article by John O. Roe, of Rochester, and is of interest to every one who uses suprarenal extract.

We are at a loss to understand why, in a book of this kind, each contribution is labeled "paper"—much on the plan of the schoolboy who labeled his drawing "this is a horse," though with less import. It would seem more in keeping with a scientific book like this, to omit such a superfluous designation.

We confess to a fondness for the transactions of this dignified association, published in a modest fashion, but always full of good material.

---

Lea's Series of Medical Epitomes. An Epitome of Physiology. For Students and Practitioners of Medicine. By Theodore C. Guenther, M. D., Assistant Physician, Norwegian Hospital, Brooklyn, and August E. Guenther, B. S., formerly Assistant in Physiology in the Medical Department, University of Michigan, Ann Arbor. Series edited by Victor C. Pedersen, A. M., M. D., Clinical Assistant in Surgery at the New York Polyclinic Medical School and Hospital. In one 12mo volume of 250 pages, with 57 engravings. Philadelphia and New York: Lea Brothers & Co. 1903. [Cloth, $1.00 net.]

In order to become a useful physician one must learn physiology, and learn it well. He must master the science from the lowest beginning point, and he must have ample laboratory teaching, as well as good textbook instruction. This excellent little work cannot supplant, but it may supplement the latter.

Indeed, that is precisely its design, which it admirably fulfils. The authors have told the essentials of the subject in an interesting manner,—concisely, succinctly, scientifically,—and it cannot fail to become a valuable aid to the study of physiology. It is more than a compend, but less than a textbook, and fills a useful place in helping one to acquire a better knowledge of the subject with which it deals.

---

The Practical Medicine Series of Year Books. Comprising ten volumes on the year's progress in medicine and surgery. Issued monthly under the general editorial charge of Gustavus P. Head, M. D., Professor of Laryngology and Rhinology in the Chicago Post-Graduate Medical School. Volume III. The Eye, Ear, Nose and Throat. Edited by Casey A. Wood, C. M., M. D., Albert H. Andrews, M. D., T. Melville Hardie, A. M., M. D. Chicago: The Year Book Publishers. 1902. [Price, $1.50; entire series, $7.50.]

The departments or topics considered in this volume are the eye, by Casey A. Wood; the ear, by Albert H. Andrews, and the nose and throat, by T. Melville Hardie. The advances in these several branches of medicine have been studiously recorded by competent observers, and the literature of the year has been well gleaned for the purpose of grouping in a single book, all the really valuable suggestions that clinical research or experimental work has brought out.

In the article on the ear, Dr. Renner's paper, published in this journal, June, 1902, is not only referred to, but liberally quoted. The methods of diagnosis and treatment of the diseases of the ear have improved very much in recent years, as will be observed by examining this work. It is as complete in all its topics as it could be made, and well repays the investment.

---

The Johns Hopkins Hospital Reports. Volume X. Nos. 6, 7, 8, 9. Baltimore: The Johns Hopkins Press. 1902.

This volume of reports deals with metabolism in albuminuria, by Charles P. Emerson; regenerative changes in the liver after acute yellow atrophy, by W. G. MacCullum; surgical features of typhoid fever, by Thomas McCrae and James F. Mitchell; and the symptoms, diagnosis and surgical treatment of ureteral calculus, by Benjamin R. Schenck. Dr. Emerson's study of the metabolic changes in albuminuria is an interesting contribution to the literature of an important subject. The other monographs, too, are scholarly contributions to their several topics. A more substantial binding would be in better harmony with the excellent presswork and paper of these reports.

---

Transactions of the American Gynecological Society. Twenty-seventh annual meeting held at Atlantic City, May 27–29, 1902. J. Riddle Goffe, M. D., Secretary. Philadelphia: William J. Dornan, Printer. 1902.

The material in this volume is interesting and points to progress in the history of the society. The changes in the personnel of the organisation, as shown by the lists, have been many in twenty years,—death, resignation, and other casualties having wrought the usual modification. New members, however, have been added from time to time, keeping the working force still strong.

The papers in this book cover many interesting portions of the field of gynecology, and will be read with profit by physicians outside the membership of the society. A contribution of special interest is that of Thomas A. Ashby, of Baltimore, relating to spleenectomy for wandering spleen. Many other papers of value go to make up an interesting book.

---

Lea's Series of Medical Epitomes. A Manual of Obstetrics for Students and Practitioners. By W. P. Manton, M. D., Adjunct Professor of Obstetrics and Professor of Clinical Gynecology, Detroit College of Medicine. In one 12mo volume of 265 pages, with 82 illustrations. Philadelphia and New York: Lea Bros & Co. 1903. [Cloth, $1.00.]

The author of this number of the epitome series is to be credited with having done well in condensing a comprehensive subject, without inflicting violence to its essentials. Only a person of experience in teaching and writing could accomplish this result. This book will serve to aid a person who already understands the subject, to refresh his memory on forgotten details. This is its real object, rather than to act as a foundation for the study of obstetrics. An undergraduate may use it in preparing for faculty examinations, and a candidate may read it with advantage in preparation for state licensing examinations. It contains many excellent engravings, and is a commendable epitome of the topic of obstetrics.

---

Proceedings of the American Medico-Psychological Association at the Fifty-eighth Annual Meeting, held at Montreal, P. Q., June 17–20, 1902. C. B. Burr, Secretary. Published by American Medico-Psychological Association.

Although this association is one of the largest of the special medical societies, before which a number of valuable papers are read, it has seen fit to publish its transactions bound in paper. This is unfortunate, for such books become lost and are seldom used for reference. The material it contains, however, is excellent.

